# Hearing Loss is Associated With Risk of Alzheimer’s Disease: A Case-Control Study in Older People

**DOI:** 10.2188/jea.JE20140147

**Published:** 2015-08-05

**Authors:** Shih-Chang Hung, Kuan-Fu Liao, Chih-Hsin Muo, Shih-Wei Lai, Chia-Wei Chang, Hung-Chang Hung

**Affiliations:** 1 Department of Emergency Medicine, Nantou Hospital, Nantou, Taiwan; 2 Graduate Institute of Integrated Medicine, China Medical University, Taichung, Taiwan; 3 Department of Internal Medicine, Taichung Tzu Chi General Hospital, Taichung, Taiwan; 4 Department of Public Health, China Medical University, Taichung, Taiwan; 5 Management Office for Health Data, China Medical University Hospital, Taichung, Taiwan; 6 School of Medicine, China Medical University, Taichung, Taiwan; 7 Department of Family Medicine, China Medical University Hospital, Taichung, Taiwan; 8 Department of Neurology, Nantou Hospital, Nantou, Taiwan; 9 Department of Internal Medicine, Nantou Hospital, Nantou, Taiwan

**Keywords:** Alzheimer’s disease, head injury, depression, hearing loss, Parkinson’s disease

## Abstract

**Background:**

It remains unknown whether hearing loss increases the risk of Alzheimer’s disease. This study aimed to examine the association between hearing loss and risk of Alzheimer’s disease in older people in Taiwan.

**Methods:**

Analyzing the database from Taiwan’s National Health Insurance Program, this case-control study enrolled 488 subjects ≥65 years old with newly diagnosed Alzheimer’s disease as a case group and 1952 subjects without Alzheimer’s disease as a control group from 1998–2011. Patients with Alzheimer’s disease and other comorbidities were identified by analyzing ICD-9 coding in claims data. The association of hearing loss, other comorbidities, and risk of Alzheimer’s disease were compared between groups.

**Results:**

After controlling for confounders, multivariable logistic regression showed an adjusted odds ratio of Alzheimer’s disease of 1.39 in people with hearing loss (95% CI, 1.05–1.84) versus those without. Parkinson’s disease (OR 4.44; 95% CI, 2.54–7.78), head injury (OR 2.31; 95% CI, 1.46–3.66), depression (OR 1.68; 95% CI, 1.19–2.39), hypertension (OR 1.40; 95% CI, 1.10–1.79), and age (each year, OR 1.03; 95% CI, 1.01–1.05) also showed strong links with Alzheimer’s.

**Conclusions:**

Hearing loss is associated with increased risk of Alzheimer’s disease in older people in Taiwan.

## INTRODUCTION

Dementia is not only a major health issue but also an ongoing non-negligible socioeconomic problem. A meta-analysis by Prince et al tallied global prevalence at 5%–7%, with around 35.6 million people afflicted in 2010.^1^ Wimo et al estimated that the worldwide costs of dementia were $604 billion (United States Dollars) in 2010, and costs are expected to rise in coming years.^2^ Caregiver burden for patients with dementia also warrants multidimensional solutions.^3^ Alzheimer’s remains the most common type of dementia in both Western and Asian populations, accounting for about 11% of Americans aged 65 years and older.^4^

In Japan, the ratio of Alzheimer’s disease to vascular dementia seems to be rising over time.^5^ Even though the pathophysiology of Alzheimer’s disease was identified as related to amyloid deposition in the brain and associated neurodegenerative processes, the mechanism of Alzheimer’s disease has not been fully elucidated. Other epidemiologic associations have been reported in recent decades.^6^^,^^7^ Most comorbidities cited as linked with Alzheimer’s also relate to aging.

Sensory impairment, such as hearing loss, is likewise a thorny problem for the elderly. Presbycusis (age-related hearing loss) usually occurs as gradual, symmetric hearing loss in the elderly. Prevalence has grown steadily with age in America; Nash et al reported a prevalence of 42.7% at ages 65–84 years in 2005–2008.^8^ Other population estimates are 25% at ages 65–75 and 70%–80% beyond age 75.^9^ The association between hearing loss and Alzheimer’s disease has been discussed since the 1980s. However, most studies have been based on Western populations. Both Alzheimer’s disease and hearing loss are associated with age. Since the prevalence of Alzheimer’s disease tended to increase in both Western and Asian populations, we wish to prove the association of hearing loss and Alzheimer’s disease in Asians.

This study evaluates the relationship of hearing loss and Alzheimer’s disease among the elderly using National Health Insurance claims data in Taiwan.

## MATERIALS AND METHODS

### Data sources

This case-control study analyzed the database of the Taiwan National Health Insurance Program, the details of which have been documented in previous studies.^10^^–^^12^ In brief, National Health Insurance began in March 1995 and covers 99% of the 23 million residents of Taiwan.^13^ The insurance covers outpatient and inpatient services. The Institutional Review Board of China Medical University and Hospital (CMU-REC-101-012) approved this study.

### Participants

According to the Diagnostic and Statistical Manual of Mental Disorders (DSM) IV definition, patients with Alzheimer’s disease are characterized by disability in daily activity function. However, it would be impossible to re-inspect the diagnostic accuracy of each case by DSM IV criteria in the nationwide insurance claims dataset. Therefore, we selected cases based on reported International Classification of Diseases 9th Revision Clinical Modification (ICD-9) codes as a substitute. Subjects aged 65 years or older newly diagnosed with Alzheimer’s disease according to ICD-9 code 331.0 in 1998–2011 were identified as the case group. The index date for each case was defined as the date of diagnosis. To prevent entanglements with unknown-type dementia and to explore the association between Alzheimer’s disease and comorbidities, strict criteria were adopted to select patients who were diagnosed with Alzheimer’s disease. Patients who were diagnosed with mixed- or unknown-type dementia or vascular dementia were all excluded from the case group. At the same time, to increase statistical power, for each case we randomly selected four subjects without Alzheimer’s as a control group. The groups were frequency matched for sex, age (within 5 years), and index date. For each Alzheimer’s disease case identified on a certain index date, there were four subjects of the same sex and age (within 5 years) selected as controls on the same day. Subjects who were assigned to the control group were free from all types of dementia in the dataset.

### Comorbidities potentially associated with Alzheimer’s disease

Other medical conditions potentially associated with Alzheimer’s disease included hearing loss (ICD-9 codes 388.01, 389.0, 389.1, 389.2, 389.8, 389.9), cerebrovascular disease (ICD-9 codes 430–438), chronic kidney disease (ICD-9 codes 585–586, 588.8–588.9), depression (ICD-9 codes 296.2–296.3, 300.4, 311), diabetes mellitus (ICD-9 codes 250), head injury (ICD-9 codes 850–854, 959.01), hypertension (ICD-9 codes 401–405), hyperlipidemia (ICD-9 codes 272.0, 272.1, 272.2, 272.3, 272.4) and Parkinson’s disease (ICD-9 codes 332.0). Comorbidities of each subject were all identified before the index date or matched index date. As mentioned above, to prevent biased results, subjects with other types of dementia (ICD-9 codes 290.0–290.4, 294.1), as well as specific noise-induced or other type of hearing loss (ICD-9 codes 388.02, 388.1, 388.2, 389.7), before the index date were excluded. Even though we adopted strict criteria to identify Alzheimer’s disease by using ICD-9 codes in insurance claims data, there may have been some degree of misclassification of dementia and miscoded comorbidities.

### Statistical analysis

We contrasted sex, age, and co-morbidities between groups using the Chi-square test for categorical variables; significant associations found in crude analysis were further included in multivariable logistic regression to measure odds ratios (ORs) and 95% confidence intervals (CIs) for risk of Alzheimer’s disease. Because controls were frequency matched, we used an unconditional logistic regression model to estimate the risk for Alzheimer’s disease.^14^ Statistical significance was set at *P* < 0.05 (SAS software version 9.1; SAS Institute Inc., Cary, NC, USA).

## RESULTS

### Characteristics of study population

In our dataset from 1988 to 2011, a total of 488 newly coded Alzheimer’s disease subjects met the antecedent criteria as cases, and 1952 subjects without Alzheimer’s disease were matched as controls. Table 1 shows intergroup demographic traits and co-morbidities. The case group had significantly higher proportion of hearing loss, depression, diabetes mellitus, head injury, hypertension, hyperlipidemia, and Parkinsonism. Mean age (standard deviation [SD]) was 76.20 (5.16) years for cases and 75.24 (5.36) years for controls. The mean (SD) follow-up duration between the diagnosis of hearing impairment and Alzheimer’s disease was 5.20 (3.50) years.

**Table 1.** Characteristics between Alzheimer’s disease cases and control subjects

	Alzheimer’s disease				*P* value^a^

	Yes *n* = 488		No *n* = 1952		
	
	*n*	(%)	*n*	(%)	
Sex					1.00
Male	216	44.26	864	44.26	
Female	272	55.74	1088	55.74	
Age group, years					1.00
65–74	193	39.55	772	39.55	
75–84	295	60.45	1180	60.45	
Co-morbidities before index date^b^					
Hearing loss	87	17.8	239	12.2	0.001
Cerebrovascular disease	63	12.91	242	12.40	0.80
Chronic kidney disease	32	6.56	109	5.58	0.41
Depression	59	12.09	117	5.99	<0.0001
Diabetes mellitus	152	31.15	474	24.28	0.002
Head injury	34	6.97	52	2.66	<0.0001
Hypertension	374	76.64	1287	65.93	<0.0001
Hyperlipidemia	133	27.25	438	22.44	0.02
Parkinson’s disease	31	6.35	24	1.23	<0.0001

^a^Chi-square test comparing subjects with and without Alzheimer’s disease.

^b^Index date was defined as date of diagnosing Alzheimer’s disease.

### Association between Alzheimer’s disease and hearing loss and/or other comorbidities

After controlling for confounders, multivariable logistic regression analysis showed an adjusted odds ratio of Alzheimer’s disease of 1.39 in subjects with hearing loss (95% CI, 1.05–1.84) compared to those without. Parkinsonism (OR 4.44; 95% CI, 2.54–7.78), head injury (OR 2.31; 95% CI, 1.46–3.66), depression (OR 1.68; 95% CI, 1.19–2.39), hypertension (OR 1.40; 95% CI, 1.10–1.79) and age (per 1 year increment, OR 1.03; 95% CI, 1.01–1.05) were associated with Alzheimer’s disease (Table 2).

**Table 2.** Odds ratios of Alzheimer’s disease associated with hearing loss and other co-morbidities

Variable	Crude		Adjusted^a^	
	
	OR	(95% CI)	OR	(95% CI)
Sex (male vs female)	1.00	(0.82–1.22)	—	
Age (per 1 year increment)	1.04	(1.02–1.06)	1.03	(1.01–1.05)
Co-morbidities before index date				
Hearing loss	1.56	(1.19–2.04)	1.39	(1.05–1.84)
Cerebrovascular disease	1.05	(0.78–1.41)	—	
Chronic kidney disease	1.19	(0.79–1.78)	—	
Depression	2.16	(1.55–3.00)	1.68	(1.19–2.39)
Diabetes mellitus	1.41	(1.14–1.75)	1.23	(0.98–1.55)
Head injury	2.74	(1.76–4.27)	2.31	(1.46–3.66)
Hypertension	1.70	(1.35–2.13)	1.40	(1.10–1.79)
Hyperlipidemia	1.30	(1.03–1.62)	1.08	(0.84–1.37)
Parkinson’s disease	5.45	(3.17–9.37)	4.44	(2.54–7.78)

CI, confidence interval; OR, odds ratio.

^a^Adjusted for age, hearing loss, depression, diabetes mellitus, head injury, hyperlipidemia, hypertension, and/or Parkinson’s disease.

## DISCUSSION

The present nationwide, population-based case-control study elucidated the association between Alzheimer’s disease and hearing loss and other comorbidities, including depression, head injury, hypertension, and Parkinson’s disease. Given the limitations of claims data, we designed this study to show the association between hearing loss and Alzheimer’s disease. Even with a relatively low number of identified cases, the statistical significance remained in our study.

Hearing impairment, including that which occurs in elderly patients, is usually designated as sensorineural, conductive, or mixed. Conductive hearing impairment arises from physical injury to the mechanical barriers that transmit sound waves to the inner ear and stimulate hearing, such as ossicle injuries and tympanic membrane sclerosis. Sensorineural hearing impairment is caused by inner ear, cochlea, or auditory nerve dysfunction. The term presbycusis chiefly describes sensorineural hearing loss in the elderly, which is noise-induced hearing loss characterized mainly by loss in high frequencies. Still, presbycusis may present as multifactorial and complicated hearing dysfunction. Patients with hearing loss obviously identified as noise-induced were excluded from our study, which found a significant association of hearing loss with Alzheimer’s disease. This association has been discussed for decades. In 1986, an observation study conducted by Uhlmann et al enrolled 156 Alzheimer’s cases and cited hearing loss as foreshadowing cognitive dysfunction.^15^ In 1989, after controlling for age, sex, and education status, they reported that hearing loss was significantly and independently linked with severity of cognitive dysfunction in an Alzheimer’s case-control study.^16^ While ever more studies have reported an association between hearing loss and Alzheimer’s disease, dementia, and cognitive dysfunction, the mechanisms and/or causal relationship of hearing loss and Alzheimer’s disease remains unknown.^17^

Gates et al reported central auditory dysfunction as a harbinger of Alzheimer’s disease.^18^ A magnetic resonance brain image study by Lin et al indicated hearing loss correlated with accelerated atrophy of the whole brain, especially the right temporal lobe.^19^ Alzheimer’s, a neurodegenerative disease, may be linked to sensorineural versus conductive hearing loss, yet without environmental enrichment, decreased stimulation in conductive hearing loss among the elderly might contribute to Alzheimer’s disease.^20^ Genetically, apolipoprotein E epsilon 4 allele may affect the rate of cognitive decline but is under-represented in nonsyndromic sensorineural hearing loss among adults.^21^^,^^22^

Factors significantly associated with Alzheimer’s disease include depression, head injury, Parkinsonism, and hypertension. Metabolic syndrome and vascular factors were seen as associated with Alzheimer’s disease in prior studies; however, while diabetes mellitus and hyperlipidemia showed potential associations before, we statistically identified only hypertension as an independent factor, possibly due to the smaller sample size of our study compared to a previous study conducted via health insurance claims data in Taiwan.^23^ In our study, to verify the correlation with hearing loss in the elderly, Alzheimer’s disease was identified rigorously, and other unknown types of dementia were excluded.

Like Alzheimer’s, Parkinson’s disease is usually thought of as a neurodegenerative condition in the elderly. We also assume an association between these two conditions, as well as between Alzheimer’s and depression, based on this epidemiologic study. However, the causal relationship remains complicated due to difficulty in assessing these clinical conditions.^24^ Terada et al assessed regional cerebral blood flow change in Alzheimer’s cases with depressive symptoms via image assitant.^25^ Connections among neuroinflammation, neurodegeneration, and depression have been discussed,^26^ which concur with prior studies reporting head injury as a risk factor of Alzheimer’s disease.^27^

As far as we know, the present study is the first population study focused on the association between Alzheimer’s disease and hearing loss, although some limitations warrant mention. First of all, an innate information bias may exist, due to the use of data from insurance claims. Using ICD-9 coding as an expedient method of confirming each participant’s diagnosis process may introduce information bias and threaten interpretability of the results. Diagnoses of the clinical conditions of interest, along with other comorbidities, were confirmed by clinical diagnostic coding, and patients with such diseases who did not make use of National Health Insurance would not be included. In addition, hearing loss patients might visit physicians more frequently. Therefore, the risk for Alzheimer’s disease could be somewhat overestimated. However, in the regression model, patients with cerebrovascular disease, chronic kidney disease, and diabetes mellitus, who may also make more frequent use of health insurance, were not at significantly increased risk for Alzheimer’s disease. In addition, the high rate of NHI coverage and high accessibility to medical service in Taiwan make our analysis more reliable. We had no genetic or biomarker testing results in the database, so we could not assess the disparity between probable and possible Alzheimer’s according to recommendations from the National Institute on Aging-Alzheimer’s Association workgroups and DSM V.^28^ Data on educational attainment, occupational attainment, and socioeconomic status, which may also be associated with hearing loss and Alzheimer’s disease, were not available in our dataset.^29^^–^^31^ The severity of hearing loss could not be rated in each subject in our study; as such, whether a dose-response relationship exists between severity of hearing loss and cognitive disabilities in Alzheimer’s disease remains unclear.

Both presbycusis and Alzheimer’s disease might develop insidiously. Using claims data, we figured that older patients who were diagnosed with hearing impairment might have higher probability of being diagnosed with Alzheimer’s disease later. At the same time, we calculated that the mean (SD) follow-up duration between the diagnosis of hearing impairment and Alzheimer’s disease was 5.20 (3.50) years. Still, we could not determine whether hearing loss was a risk factor, precursor, or possible non-amnestic criterion of Alzheimer’s disease. Further large-scale studies of comprehensive hearing tests combined with rigorous evaluation of Alzheimer’s diagnoses and nuclear brain imaging might be able to more robustly assess the association of hearing loss and Alzheimer’s disease in older people.

In conclusion, hearing loss is associated with increased risk of Alzheimer’s disease in older people in Taiwan. Whether a non-memory-related feature of Alzheimer’s disease or not, the association warrants further exploration.
